# Systolic Blood Pressure and Microaxial Flow Pump–Associated Survival in Infarct-Related Cardiogenic Shock: A Post Hoc Analysis of the DanGer Shock Randomized Clinical Trial

**DOI:** 10.1001/jamacardio.2025.3337

**Published:** 2025-08-30

**Authors:** Astrid Duus Mikkelsen, Rasmus Paulin Beske, Lisette Okkels Jensen, Hans Eiskjær, Norman Mangner, Amin Polzin, Christian Schulze, Carsten Skurk, Peter Nordbeck, Benedikt Schrage, Vasileios Panoulas, Sebastian Zimmer, Andreas Schäfer, Thomas Engstrøm, Lene Holmvang, Martin Frydland, Anders Bo Junker, Henrik Schmidt, Nanna Louise Junker Udesen, Kristian Wachtell, Christian Juhl Terkelsen, Axel Linke, Jesper Kjærgaard, Jacob Eifer Møller, Christian Hassager

**Affiliations:** 1 Department of Cardiology, Copenhagen University Hospital Rigshospitalet, Denmark; 2 Department of Cardiology, Odense University Hospital, Odense, Denmark; 3 Department of Cardiology, Aarhus University Hospital, Aarhus, Denmark; 4 Department of Internal Medicine and Cardiology, Heart Centre Dresden, Dresden, Germany; 5 Department of Cardiology, Pulmonology, and Vascular Medicine, University Hospital Düsseldorf, Medical Faculty of the Heinrich Heine University Düsseldorf, and the Cardiovascular Research Institute Düsseldorf, Germany; 6 Department of Internal Medicine I, Cardiology, Angiology, and Intensive Medical Care, University Hospital Jena, Jena, Germany; 7 Campus Benjamin Franklin, and Deutsches Zentrum für Herz Kreislauf Forschung, Berlin, Germany; 8 Department of Internal Medicine I, University Hospital Würzburg, Würzburg, Germany; 9 Department of Cardiology, University Heart and Vascular Centre Hamburg, Hamburg, Germany; 10 Royal Brompton and Harefield Hospitals, Guy’s and St Thomas’ NHS Foundation Trust, Harefield Hospital, Harefield, United Kingdom.; 11 Department of Cardiology, University Hospital Bonn, Bonn, Germany; 12 Department of Cardiology and Angiology, Hannover Medical School, Hanover, Germany; 13 Department of Clinical Medicine, University of Copenhagen, Copenhagen, Denmark; 14 Department of Anesthesiology and Intensive Care Medicine, Odense University Hospital, Odense, Denmark

## Abstract

**Importance:**

Microaxial flow pump treatment improves survival in selected patients with infarct-related cardiogenic shock; however, treatment carries substantial risks, and benefit may vary by patient subgroup. Systolic blood pressure (SBP) has been proposed as a modifier of the survival benefit.

**Objective:**

To investigate whether SBP at randomization modifies the survival benefit of microaxial flow pump treatment in ST-segment elevation myocardial infarction–related cardiogenic shock.

**Design, Setting, and Participants:**

This was a post hoc analysis of the Danish-German (DanGer) Shock open-label randomized clinical trial among adult patients with ST-segment elevation myocardial infarction complicated by cardiogenic shock, conducted between 2013 and 2023 at 14 tertiary invasive cardiac centers in Denmark, Germany, and the United Kingdom. Data analysis was performed from January 7 to April 7, 2024.

**Intervention:**

Microaxial flow pump therapy plus standard care vs standard care alone.

**Main Outcomes and Measures:**

All-cause mortality at 180 days according to randomization SBP.

**Results:**

Of 355 patients included in the DanGer Shock trial, 351 patients had available SBP at randomization (median [IQR] age, 69 [59-76] years; 277 [79%] male). In a dichotomized regression analysis, microaxial flow pump treatment significantly reduced mortality for SBPs lower than 82 mm Hg compared with standard care alone (odds ratio [OR], 0.34; 95% CI, 0.18-0.63; *P* < .001). This was not evident for higher pressures (OR, 0.96; 95% CI, 0.53-1.70; *P* = .90; *P* for interaction = .02). Kaplan-Meier survival analysis and spline regression analysis supported these findings (*P* for interaction = .02; *P* for nonlinearity = .01).

**Conclusions and Relevance:**

Randomization SBP was associated with the survival benefit of microaxial flow pump treatment, with the most hypotensive patients deriving the largest survival benefit. Early SBP may help identify patients most likely to gain a net benefit from microaxial flow pump treatment. Findings are hypothesis generating.

**Trial Registration:**

ClinicalTrials.gov Identifier: NCT01633502

## Key Points

**Question:**

Does systolic blood pressure (SBP) modify the survival benefit of microaxial flow pump therapy in ST-segment elevation myocardial infarction–related cardiogenic shock?

**Findings:**

In this post hoc analysis of the DanGer Shock randomized clinical trial including 351 patients, SBP at randomization significantly modified the survival benefit of microaxial flow pump therapy, with benefit most pronounced in patients with the lowest SBPs. Adverse events occurred independently of SBP at randomization.

**Meaning:**

Early SBP may help identify patients most likely to derive a net benefit from microaxial flow pump treatment in infarct-related cardiogenic shock.

## Teaser

This post hoc analysis of the DanGer Shock randomized clinical trial investigates whether systolic blood pressure at randomization modifies the survival benefit of microaxial flow pump treatment in ST-segment elevation myocardial infarction–related cardiogenic shock.

## Introduction

Cardiogenic shock (CS) remains a frequent complication of acute myocardial infarction (AMI), carrying a poor prognosis with short-term mortality rates of 40% to 50%.^1,2,3^ AMI-CS pathophysiology is characterized by inadequate tissue perfusion due to an acutely failing heart.^4^ As a marker of hemodynamic compromise, hypotension is a central diagnostic criterion and serves as a standard treatment target.^5,6,7^ After nearly a decade of sparse progression in evidence-based AMI-CS treatment,^3,6,8^ the Danish-German (DanGer) Shock randomized clinical trial demonstrated a significant reduction in 180-day mortality with routine use of a microaxial flow pump in selected patients with ST-segment elevation myocardial infarction (STEMI) complicated by CS (STEMI-CS).^9^ However, the survival benefit came with an increased risk of complications, such as moderate or severe bleeding, limb ischemia, acute kidney injury (AKI), and stroke.^9^ Subgroup analysis of the DanGer Shock trial, as well as emerging evidence, has suggested specific patient subgroups with altered risk-benefit profiles.^9,10^ A recent individual patient data meta-analysis suggested an interaction between systolic blood pressure (SBP) at randomization and the survival effect of mechanical circulatory support (MCS).^11^

In this post hoc analysis of the DanGer Shock trial, we conduct a comprehensive analysis of the impact of SBP at randomization on 180-day all-cause mortality, on microaxial flow pump–associated survival benefit, and on risk of adverse treatment events.

## Methods

### Study Design

This is an exploratory, post hoc analysis of the DanGer Shock trial, a multicenter, randomized, open-label clinical trial conducted at 14 tertiary invasive cardiac centers in Denmark, Germany, and the United Kingdom investigating the survival benefit of transvalvular microaxial flow pump treatment in patients with STEMI-CS.^9^ The DanGer Shock trial design has been published elsewhere.^9^ In brief, eligible patients were randomly assigned to receive treatment with a microaxial flow pump (Impella CP; Abiomed/Johnson & Johnson MedTech) plus standard guideline-directed therapy vs standard guideline-directed therapy alone. In accordance with applicable legislation, informed consent was obtained as soon as possible in a stepwise manner—first via proxy (next of kin or independent physician), then directly from the patient when possible. The trial protocol was approved by the ethics committees of all participating sites and has been published elsewhere.^12,13^ Data analysis was performed from January 7 to April 7, 2024.

### Study Population

The study included patients aged 18 years or older who presented with STEMI and CS. CS was defined as hypotension (SBP <100 mm Hg or need for vasopressors), hypoperfusion (lactate level >22.52 mg/dL [to convert to millimoles per liter, multiply by 0.111]), and left ventricular dysfunction (left ventricular ejection fraction [LVEF] <45%). Patients resuscitated from out-of-hospital cardiac arrest with a persistent Glasgow Coma Scale score less than 8, noncardiogenic shock, significant aortic valve stenosis or regurgitation, mechanical complications of the myocardial infarction, or right ventricular failure were excluded from trial participation. The full inclusion and exclusion criteria have been published previously.^9^ Only patients with recorded SBP at randomization were included in the present analysis.

### Treatment Protocol and Intervention

Eligible patients were randomized immediately on diagnosis of shock. This could occur in the catheterization laboratory and up to 12 hours after revascularization. In the catheterization laboratory, patients underwent emergency revascularization of the culprit coronary lesion and received vasopressor therapy in concordance with local guidelines. If assigned, the microaxial flow pump was implanted immediately after randomization and run at the highest possible performance level for a minimum of 48 hours. During the subsequent cardiac intensive care unit (CICU) admission, patients were managed according to predefined hemodynamic treatment goals, including mean arterial pressure (MAP) higher than 65 mm Hg, cardiac index higher than 2.0 L/min/m^2^, mixed venous oxygen saturation (Svo_2_) higher than 55%, and arterial lactate level less than 22.52 mg/dL. In the event of hemodynamic instability, treatment could be escalated to further MCS at the discretion of the treating shock team. Full details of treatment goals, MCS escalation, and weaning from the microaxial flow pump have been reported elsewhere.^12,13^

### Systolic Blood Pressure

The randomization SBP central to the present analysis was obtained at study randomization. It was measured invasively during coronary angiography in the catheterization laboratory or via arterial cannula reading in the CICU. Patients were arbitrarily divided into quartiles of SBP for group comparison.

### Vasoactive-Inotropic Score

The Vasoactive-Inotropic Score (VIS) was calculated for predefined time points during the first 12 hours of CICU stay (CICU admission, 1 hour, 3 hours, 6 hours, and 12 hours). The VIS quantifies the intensity of pharmacologic cardiovascular support in terms of vasopressors and inotropic agents and is calculated as dopamine dose (micrograms per kilogram per minute) + dobutamine dose (micrograms per kilogram per minute) + 100 × epinephrine dose (micrograms per kilogram per minute) + 10 × milrinone (micrograms per kilogram per minute) + 100 × norepinephrine dose (micrograms per kilogram per minute) + 50 × levosimendan dose (micrograms per kilogram per minute).^14^ The VIS at randomization was not recorded.

### End Points

The primary end point was 180-day all-cause mortality according to randomization SBP. Secondary end points comprised the MAP and VISs during the first 12 hours of CICU admission, and adverse events such as moderate or severe bleeding (according to the Global Utilization of Streptokinase and Tissue Plasminogen Activator for Occluded Arteries criteria), AKI (according to the Risk, Injury, and Failure, Sustained Loss, and End-Stage Kidney Disease classification), need for kidney replacement therapy (KRT), limb ischemia (defined as clinical signs or symptoms of impaired limb perfusion requiring vascular surgical evaluation or resulting in device explantation), and stroke.

### Statistical Analysis

Results are presented as median with interquartile range (IQR). Categorical variables are presented as numbers with percentages. Differences in characteristics between quartiles of SBP are addressed by Kruskal-Wallis rank sum test, Pearson χ^2^ test, and Fisher exact test as appropriate.

The association between randomization SBP and 180-day all-cause mortality was evaluated using logistic regression modeling. Guided by a review of the literature on independent mortality predictors in AMI-CS,^15,16,17^ we created a directed acyclic graph and constructed the following sequential models: (1) an unadjusted model; (2) a confounder-adjusted model (adjusted for preexposure confounders, including age, sex, diabetes, hypertension, prior stroke, estimated glomerular filtration rate at admission, time from symptom onset to randomization [log_2_ transformed], resuscitation prior to arrival in the catheter laboratory, number of diseased coronary vessels, and randomization group); and (3) a confounder- and mediator-adjusted model (further adjusted for potential mediators including lactate level, LVEF, Thrombolysis in Myocardial Infarction flow following percutaneous coronary intervention, blood glucose level, and Society for Cardiovascular Angiography and Interventions stage), representing an exploratory approximation of the controlled direct effect.

Randomization SBP was modeled as a continuous variable using restricted cubic splines to allow for nonlinearity. Missing data on regression model covariates (not SBP) were imputed before modeling using a nonparametric random forest approach, as implemented in the missForest package in RStudio version 6.1.524 (R Core Team).

The impact of microaxial flow pump vs standard care on 180-day mortality according to randomization SBP was investigated first by Kaplan-Meier survival curves and log-rank test across quartiles of SBP and second by logistic regression modeling including an interaction term of randomization and SBP. In the regression modeling, a dichotomous analysis was performed for randomization SBP above and below the median. Subsequently, randomization SBP was modeled as a continuous variable using restricted cubic spline regression to allow for nonlinearity. The optimal number of knots was guided by comparing the Akaike information criterion between candidate models.

The association between VIS and randomization SBP at discrete time points during the first 12 hours of CICU admission was analyzed using a repeated-measures model with an unstructured covariance matrix. VIS was square root transformed due to right skewness. Covariates included SBP (continuous), randomization group, and time (categorical). Interaction terms were tested but excluded due to nonsignificance, optimizing model fit based on Akaike information criterion.

A sensitivity analysis of the primary end point was performed investigating the randomization MAP. To account for death as a potential competing risk in adverse events incidence, a composite of the respective adverse events and death during CICU stay was analyzed.

All analyses were performed in the intention-to-treat population. A 2-sided *P* < .05 was considered statistically significant. All data management, statistical analyses, and figure generation were performed using RStudio version 6.1.524 (R Core Team).

## Results

A total of 355 patients were enrolled in the DanGer Shock trial. Four patients had no recorded randomization SBP and were excluded from the analysis—all 4 were included based on vasopressor need, and 2 of these patients died. The final study population thus comprised 351 patients (median [IQR] age, 69 [59-76] years; 277 [79%] male). Of these, 27 patients (8%) were included based on vasopressor need alone, with a randomization SBP higher than 100 mm Hg. The median (IQR) SBP was 68 (60-70) mm Hg for the lowest quartile, 79 (75-80) mm Hg for quartile 2, 89 (85-90) mm Hg for quartile 3, and 100 (95-105) mm Hg for quartile 4 (Table 1). Allocation to microaxial flow pump was evenly distributed across quartiles. Known prior hypertension was present in approximately half of the patients and was similar in all 4 quartiles (Table 1). There was no significant difference in arterial lactate levels or LVEF at randomization between quartiles. Time from symptom onset to randomization, as well as hospital admission to randomization, was longer in the higher SBP quartiles (Table 1). The timing of randomization was similar across all quartiles, with most patients being randomized during their catheterization laboratory stay.

**Table 1.** Baseline Characteristics of the Study Population

Characteristic	SBP at randomization				*P* value
	Q1 (n = 89)	Q2 (n = 90)	Q3 (n = 90)	Q4 (n = 82)	
Age, median (IQR), y	70 (60-77)	69 (59-75)	66 (59-74)	69 (59-75)	.30
Sex, No. (%)					
Female	21 (24)	16 (18)	20 (22)	17 (21)	.80
Male	68 (76)	74 (82)	70 (78)	65 (79)	
BMI, median (IQR)	26 (23-28)	26 (24-29)	27 (25-30)	27 (25-29)	.11
Comorbidity, No. (%)					
Hypertension	44 (49)	51 (57)	44 (49)	42 (51)	.70
Diabetes	12 (13)	14 (16)	29 (32)	23 (28)	.004
Previous myocardial infarction	11 (12)	16 (18)	13 (14)	14 (17)	.70
Chronic heart failure	5 (6)	10 (11)	4 (4)	9 (11)	.30
Chronic kidney disease	7 (8)	8 (9)	11 (12)	7 (9)	.80
Catheterization laboratory characteristic, No. (%)					
SCAI stage					
C	38 (43)	49 (54)	55 (61)	53 (65)	.02
D or E	51 (57)	41 (46)	35 (39)	29 (35)	
Anterior myocardial infarction	65 (73)	70 (78)	63 (70)	56 (68)	.50
PCI of culprit performed	88 (99)	87 (97)	83 (92)	81 (99)	.08
Multivessel disease on CAG	69 (78)	58 (64)	65 (72)	61 (74)	.20
VT or VF with cardioversion	32 (36)	34 (38)	22 (24)	15 (18)	.01
Randomization characteristic					
SBP, median (IQR), mm Hg	68 (60-70)	79 (75-80)	89 (85-90)	100 (95-105)	NA
Mean arterial pressure, median (IQR), mm Hg	50 (48-57)	60 (53-66)	69 (64-73)	79 (71-87)	NA
Heart rate, median (IQR), beats/min	94 (77-106)	90 (77-107)	98 (78-111)	97 (75-112)	.40
Lactate concentration, median (IQR), mg/dL	45.05 (27.03-81.08)	45.05 (36.04-63.06)	36.04 (27.03-63.06)	36.04 (27.03-54.05)	.11
LVEF, median (IQR), %	20 (15-30)	25 (15-30)	25 (20-35)	25 (20-30)	.05
Resuscitation before randomization, No. (%)	16 (18)	20 (22)	19 (21)	14 (17)	.80
Time from symptom onset to randomization, median (IQR), h	4 (3-9)	4 (2-8)	9 (4-32)	8 (3-27)	<.001
Time from hospital admission to randomization, median (IQR), min	55 (31-86)	48 (27-83)	62 (33-191)	75 (43-173)	.002
Timing of randomization, No. (%)					
Catheterization laboratory, before revascularization	42 (47)	53 (59)	62 (69)	42 (51)	.09
Catheterization laboratory, after revascularization	31 (35)	25 (28)	17 (19)	23 (28)	
≤12 h after catheterization laboratory	16 (18)	12 (13)	11 (12)	17 (21)	
Allocated to microaxial flow pump therapy, No. (%)	44 (49)	44 (49)	49 (54)	40 (49)	.90

Abbreviations: BMI, body mass index (calculated as weight in kilograms divided by height in meters squared); CAG, coronary angiography; LVEF, left ventricular ejection fraction; NA, not applicable; PCI, percutaneous coronary intervention; Q, quartile; SBP, systolic blood pressure; SCAI, Society for Cardiovascular Angiography and Interventions; VF, ventricular fibrillation; VT, ventricular tachycardia.

SI conversion factor: To convert lactate concentration to millimoles per liter, multiply by 0.111.

### Mortality by Randomization SBP

**Figure 1.**
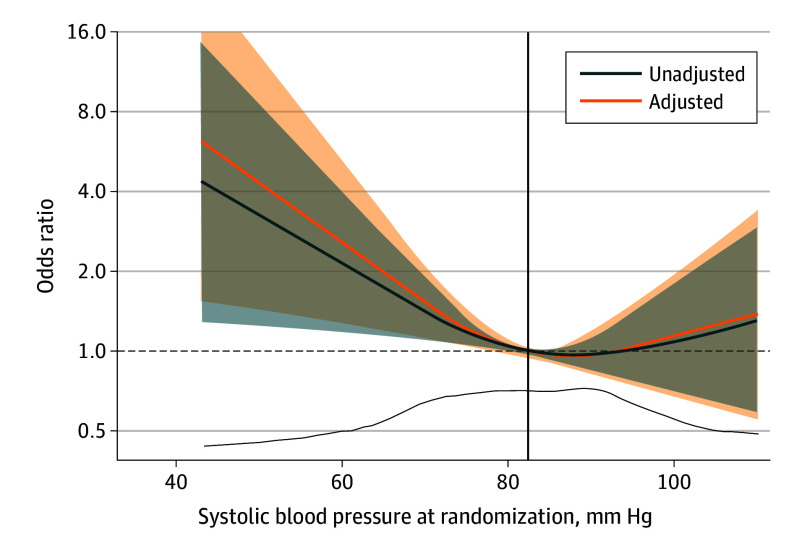
Odds Ratios for 180-Day Mortality by Systolic Blood Pressure at Randomization Odds ratios for 180-day all-cause mortality by systolic blood pressure at randomization, modeled using restricted cubic splines to allow for nonlinearity. Shaded areas indicate 95% CI. Vertical black line indicates the median systolic blood pressure; dashed horizontal line indicates an odds ratio of 1. The bottom curve indicates data density.

Randomization SBP showed a significant association with 180-day mortality (unadjusted *P* = .04; adjusted *P* = .02), with the odds of death being 4-fold higher for the lowest SBP relative to the median of 82 mm Hg in the unadjusted model (*P* = .02) and 6-fold higher in the confounder-adjusted model (*P* = .01) (Figure 1). In contrast, no association between randomization SBP and mortality was observed for higher SBPs compared with the median (Figure 1) (unadjusted *P* = .51; adjusted *P* = .49). When further adjusting for potential downstream mediators, the association between SBP and 180-day mortality was attenuated (*P* = .48) (eFigure 1 in Supplement 1). Death during percutaneous coronary intervention and death within the first day of randomization were significantly higher for the lower quartiles of randomization SBP, while MCS escalation showed no such association (Table 2).

**Table 2.** Short-Term Mortality, MCS Escalation, and Incidence of Adverse Events According to Quartiles of SBP at Randomization

Outcome	No. (%) by SBP quartile at randomization				*P* value
	Q1 (n = 89)	Q2 (n = 90)	Q3 (n = 90)	Q4 (n = 82)	
Death while in catheterization laboratory	13 (15)	5 (6)	3 (3)	2 (2)	.004
Death on day 1^a^	29 (33)	18 (20)	8 (9)	10 (12)	<.001
MCS escalation^b^	17 (19)	19 (21)	13 (14)	15 (18)	.70
Moderate or severe bleeding					
Standard care alone	4 (9)	6 (13)	5 (12)	6 (14)	NA
mAFP therapy plus standard care	7 (16)	8 (18)	13 (27)	11 (28)	
Acute kidney injury					
Standard care alone	24 (53)	21 (46)	12 (29)	21 (50)	NA
mAFP therapy plus standard care	25 (57)	26 (59)	30 (61)	27 (68)	
Kidney replacement therapy					
Standard care alone	14 (31)	9 (20)	8 (20)	15 (36)	NA
mAFP therapy plus standard care	13 (30)	20 (46)	25 (51)	15 (36)	
Limb ischemia					
Standard care alone	0	0	0	2 (5)	NA
mAFP	3 (7)	3 (7)	1 (2)	3 (8)	
Stroke					
Standard care alone	1 (2)	1 (2)	1 (2)	0	NA
mAFP therapy plus standard care	2 (5)	1 (2)	3 (3)	1 (3)	

Abbreviations: MCS, mechanical circulatory support; NA, not applicable; Q, quartile; SBP, systolic blood pressure.

^a^ Death within the first 24 hours after study randomization.

^b^ Venoarterial extracorporeal membrane oxygenation, durable left ventricular assist device, or Impella 2.5 or 5.0 (Abiomed/Johnson & Johnson MedTech).

### VIS and MAP According to Randomization SBP

During the first 12 hours of the CICU stay, all patients achieved the predefined MAP target of higher than 65 mm Hg, regardless of randomization SBP (eTable 1 in Supplement 1). VISs tended to be higher with lower randomization SBP. When stratified by quartiles, this association was not statistically significant (eTable 1 in Supplement 1). In a repeated-measures model including SBP as a continuous variable, this association proved significant, with a 1–mm Hg increase being associated with a β = 0.02 (95% CI, −0.04 to 0.00; *P* = .04) decrease in square-root VIS.

### Mortality by Randomization SBP and Microaxial Flow Pump Group

**Figure 2.**
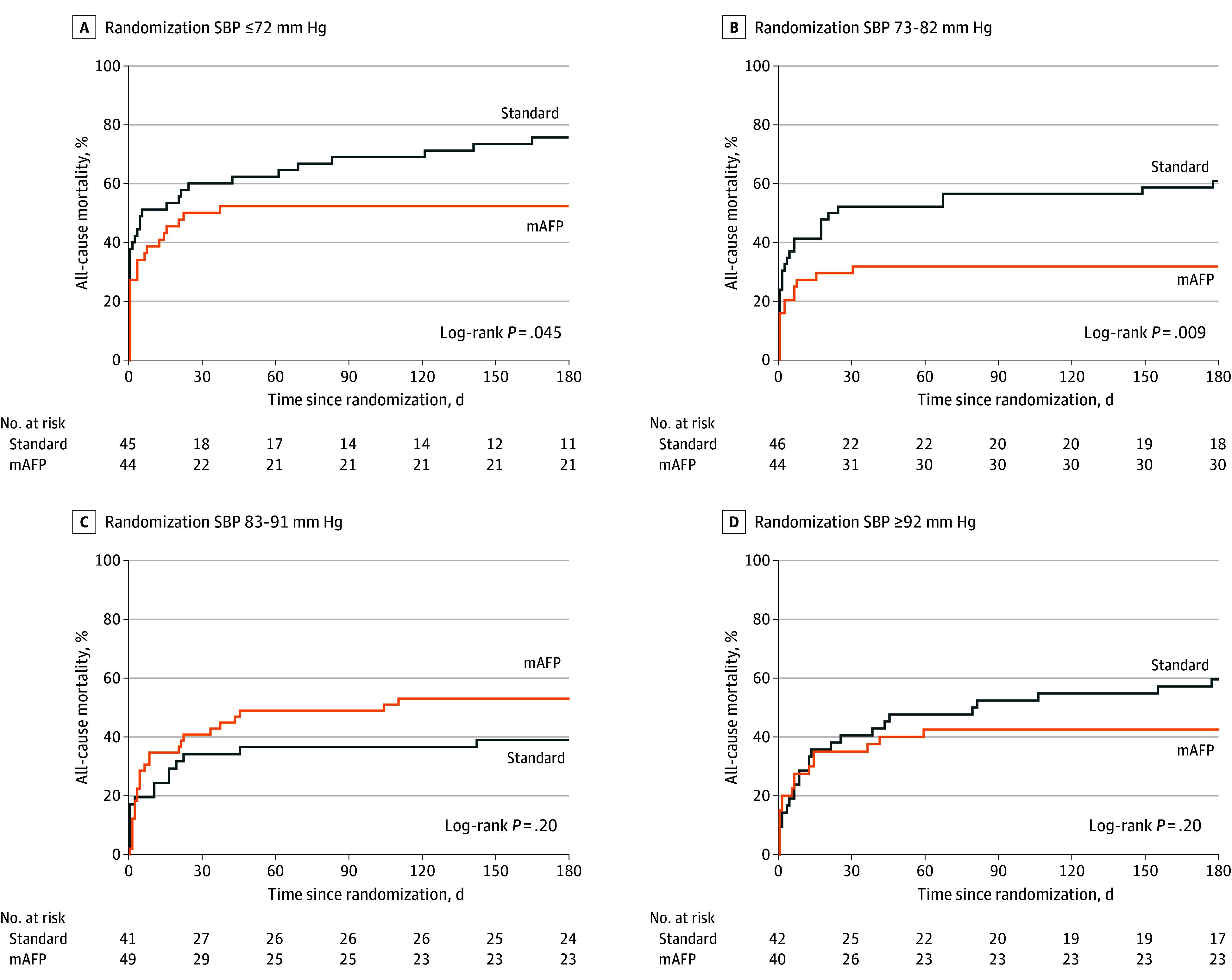
Kaplan-Meier Survival Curves for 180-Day Mortality by Treatment Group Across Quartiles of Systolic Blood Pressure at Randomization Kaplan-Meier survival curves show 180-day all-cause mortality in the group who received microaxial flow pump (mAFP) therapy plus standard care vs the group who received standard care alone within quartiles of systolic blood pressure (SBP) at randomization. Log-rank test was used to assess differences in survival between groups within each quartile.

**Figure 3.**
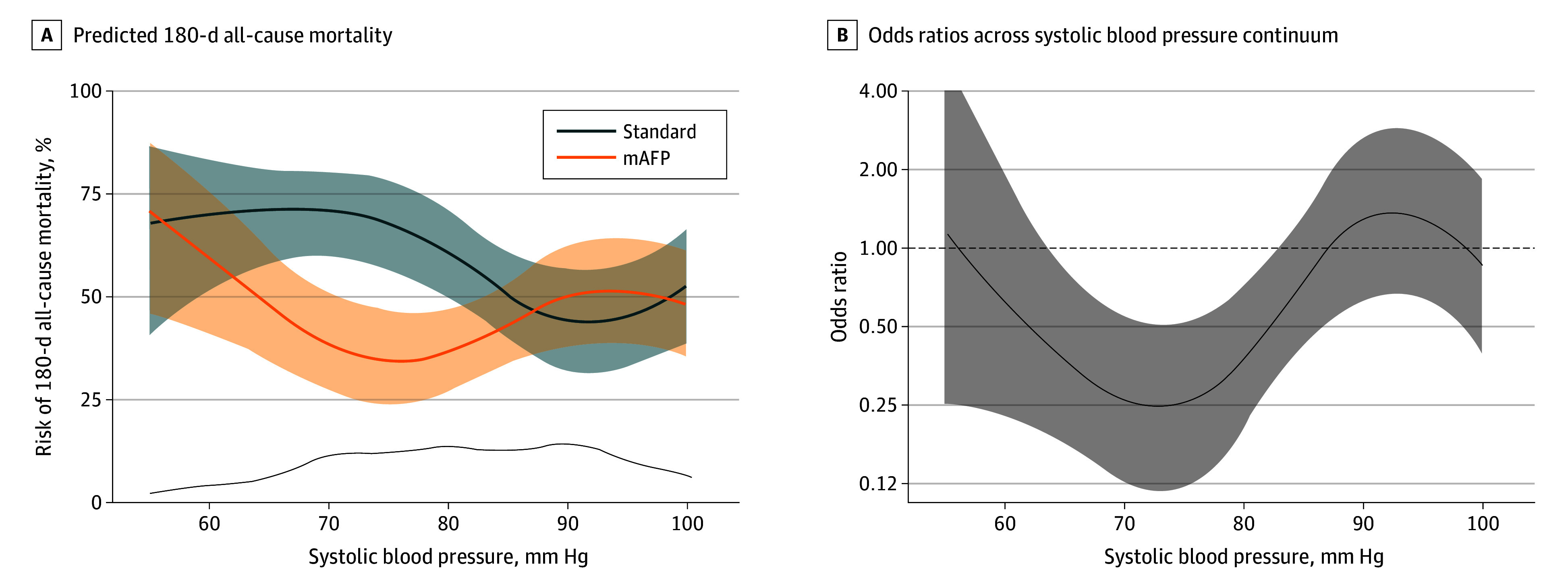
180-Day Mortality by Treatment Group Across a Continuum of Randomization Systolic Blood Pressures: Risk Estimates and Odds Ratios A, Predicted 180-day all-cause mortality in the group who received microaxial flow pump (mAFP) therapy plus standard care vs the group who received standard care alone across a continuum of systolic blood pressures at randomization. The bottom curve indicates data density. B, Corresponding odds ratios across the continuum of systolic blood pressure at randomization. Dashed line indicates an odds ratio of 1. The model is unadjusted. Shaded areas indicate 95% CI.

Among patients with a randomization SBP below the median of 82 mm Hg, microaxial pump treatment significantly reduced 180-day mortality compared with standard care alone (odds ratio [OR], 0.34; 95% CI, 0.18-0.63; *P* < .001). For randomization SBPs of 82 mm Hg or higher, no mortality difference was observed between groups (OR, 0.96; 95% CI, 0.53-1.70; *P* = .90; *P* for interaction = .02). These findings were consistent with the Kaplan-Meier survival analysis stratified by quartiles of randomization SBP (Figure 2) and further by the spline regression analysis, assessing SBP as a continuum (*P* for interaction = .02; *P* for nonlinearity = .01) (Figure 3). Findings were consistent in a sensitivity analysis investigating randomization MAP (eFigure 2 in Supplement 1).

### Adverse Events According to Randomization SBP

The incidence of moderate or severe bleeding, AKI, KRT, limb ischemia, and stroke did not differ significantly with randomization SBP (Table 2). In a sensitivity analysis, a significant interaction was observed between SBP and the composite outcome of moderate or severe bleeding or death during CICU stay (*P* for interaction = .02), although no consistent trend was evident (eTable 2 in Supplement 1). All other interactions remained nonsignificant (eTable 2 in Supplement 1).

## Discussion

In this post hoc analysis of the DanGer Shock trial, we found low SBP at randomization to be an independent predictor of 180-day all-cause mortality in patients with STEMI-CS. Second, randomization SBP significantly modified the survival benefit of microaxial flow pump therapy, with benefit most pronounced in the most hypotensive patients. Adverse treatment events, however, appeared to occur independently of randomization SBP. Our findings underscore that microaxial flow pump therapy can improve survival in subgroups of patients with AMI-CS, but careful patient selection remains essential due to the complex risk-benefit profile of this treatment. The randomization SBP may be considered, alongside age, in patient-centered decision-making for this device therapy.^10^

In AMI-CS, hypotension due to acute left ventricular failure and a decrease in cardiac output is initially maintained via a compensatory increase in peripheral vascular resistance. However, as left ventricular dysfunction progresses, blood pressure ultimately declines. SBP remains a readily obtainable clinical parameter and key component in various risk prediction scores for AMI and CS.^15,18,19,20^ Several studies have suggested low SBP as an independent predictor of mortality in AMI-CS.^6,18,21,22,23^ Conflicting evidence does exist, however.^24^ Blood pressure is not a static factor, and variations in timing of measurement, degree of vasopressor support, and particularly the immediate response to vasopressor treatment are important confounding factors that may explain the observed discrepancies. These factors are likely pivotal to both prognostic impact and clinical application. Notably, the SBPs in the present analysis were unlikely to be naive. We lack detailed information on vasoactive drug administration during catheterization laboratory stay, where the majority of patients were randomized. Here, vasoactive drugs are often given as bolus injections. Standard clinical practice in Denmark, Germany, and the United Kingdom dictates immediate initiation of vasopressor therapy in patients with profound hypotension due to AMI-CS. Thus, our SBPs likely reflect varying degrees of vasopressor-treated hypotension, including treatment-refractory cases, and should be interpreted accordingly.

Robust evidence on the influence of blood pressure on microaxial flow pump–mediated survival is scarce. Being an axial flow pump, device performance is afterload sensitive and thus depends on systemic vascular resistance and blood pressure. In a translational CS model, Udesen et al^25^ demonstrated that vasoconstriction with norepinephrine led to poorer device support and a reduction in Svo_2_. This may have influenced the present results. The current study is an extension of a recent meta-analysis by Thiele et al.^11^ This meta-analysis compiled individual patient data from 9 randomized clinical trials, including DanGer Shock, suggesting that randomization SBP below 80 mm Hg was associated with a significant 6-month survival benefit when treating patients with any MCS device, including venoarterial extracorporeal membrane oxygenation. However, the meta-analysis was not designed to investigate the present question. The prespecified subgroup analysis of the DanGer Shock trial, based on median MAP at randomization, also indicated that a survival benefit of microaxial flow pump treatment was confined to patients with a randomization MAP below the median of 63 mm Hg. However, this analysis was not powered to establish definitive effects. These data, together with our current findings, suggest a blood pressure dependency in MCS-mediated survival. However, findings remain exploratory and hypothesis generating.

Microaxial flow pump treatment carries risks. Adverse events are frequent and occurred in our analysis independent of randomization SBP. Death as a competing risk must be considered, particularly given the fact that the most hypotensive patients experience significantly higher short-term mortality rates (Table 2). However, our sensitivity analysis combining death during CICU stay with each adverse event (eTable 2 in Supplement 1) did not alter these findings. It is crucial to consider the low numbers in this stratified analysis when interpreting results. The DanGer Shock trial was not sufficiently powered to address this outcome in relation to the randomization SBP.

Our VIS and MAP data provide interesting insights into the association between blood pressure and device response. During the CICU stay, all patients, regardless of randomization SBP, achieved the target MAP greater than 65 mm Hg (eTable 1 in Supplement 1). However, the need for vasoactive support varied, with the lowest SBPs requiring a higher VIS than the higher blood pressures. Ultimately, all patients, regardless of SBP status at randomization, mobilized the relevant hemodynamic response (eTable 1 in Supplement 1). In line with our previous findings, this may suggest that low randomization SBP serves as a proxy for AMI-CS disease severity, rather than hemodynamic failure to be fixed by the microaxial flow pump. The sickest patients are thus the ones benefiting most from risky device therapy. Our data further indicate a correlation of shorter times from symptom onset and hospital admission to randomization with lower SBP (Table 1). This may suggest that early escalation to device therapy is particularly important in patients with the lowest blood pressures. However, the present data do not allow for robust conclusions.

Early SBP may offer independent prognostic information in AMI-CS not captured by lactate level. As an established predictor of mortality in AMI-CS, lactate level is widely used as a marker of microcirculatory compromise.^22,26,27^ In our study, arterial lactate levels were similar across SBP quartiles at randomization (Table 1). It must be noted that lactatemia was a trial inclusion criterion. However, our findings support the importance of a multimodal approach to early AMI-CS prognostication and device therapy decision-making, including markers of both macrocirculatory and microcirculatory compromise.

### Limitations

This study has limitations. In addition to the lack of data on vasoactive drug administration at the time of blood pressure measurement, the post hoc nature of this study means that findings are hypothesis generating. Confidence intervals have not been adjusted for multiplicity and should not be interpreted as a substitute for formal hypothesis testing. Data are sparse at the extremes of the randomization SBP range, warranting cautious interpretation of findings here. Nonmortality outcomes should also be approached carefully, as competing risks may play a significant role. While the study protocol guided MAP targets, the study was unblinded and a potential for treatment bias remains. Furthermore, we cannot account for a potential time delay between shock diagnosis and measurement of randomization SBP. Also, it must be emphasized that findings are only generalizable to patients meeting the inclusion criteria of the DanGer Shock trial—in particular, SBP below 100 mm Hg or a need for vasopressors was mandatory for study participation.

## Conclusions

This post hoc analysis of the DanGer Shock trial suggests that the survival benefit from microaxial flow pump treatment is most evident in patients with STEMI-CS with the most profound systolic hypotension. However, adverse events occurred independent of randomization SBP.

## Notes

Supplement 1. **eTable 1.** MAP and VIS During First 12 Hours of CICU Admission According to Randomization Systolic Blood Pressure **eTable 2.** Incidence of Adverse Events or Death During CICU Stay According to Quartiles of Randomization Systolic Blood Pressure **eFigure 1.** Odds Ratios for 180-Day Mortality by Randomization Systolic Blood Pressure **eFigure 2.** 180-Day Mortality by Treatment Group Across Mean Arterial Pressure at Randomization

Supplement 2. DanGer Shock Investigators

Supplement 3. Data Sharing Statement
