# Supplementary material for: Systolic Blood Pressure and Microaxial Flow Pump–Associated Survival in Infarct-Related Cardiogenic Shock: A Post Hoc Analysis of the DanGer Shock Randomized Clinical Trial
Source: JAMA Cardiol. 2025 Aug 30;10(11):1157–65. doi: 10.1001/jamacardio.2025.3337 (PMC12398770; doi:10.1001/jamacardio.2025.3337)
Supplement: Supplement 1. — eTable 1. MAP and VIS During First 12 Hours of CICU Admission According to Randomization Systolic Blood Pressure eTable 2. Incidence of Adverse Events or Death During CICU Stay According to Quartiles of Randomization Systolic Blood Pressure eFigure 1. Odds Ratios for 180-Day Mortality by Randomization Systolic Blood Pressure eFigure 2. 180-Day Mortality by Treatment Group Across Mean Arterial Pressure at Randomization [file jamacardiol-e253337-s001.pdf]

Supplemental Online Content

Mikkelsen AD, Beske RP, Jensen LO, et al; DanGer Shock Investigators. Systolic blood pressure and microaxial flow pump–associated survival in infarct-related cardiogenic shock: a post hoc analysis of the DanGer Shock randomized clinical trial. *JAMA Cardiol*. Published online August 30, 2025. doi:10.1001/jamacardio.2025.3337

eTable 1. MAP and VIS During First 12 Hours of CICU Admission According to Randomization Systolic Blood Pressure
eTable 2. Incidence of Adverse Events or Death During CICU Stay According to Quartiles of Randomization Systolic Blood Pressure
eFigure 1. Odds Ratios for 180-Day Mortality by Randomization Systolic Blood Pressure
eFigure 2. 180-Day Mortality by Treatment Group Across Mean Arterial Pressure at Randomization

This supplemental material has been provided by the authors to give readers additional information about their work.

© 2025 Mikkelsen AD et al. *JAMA Cardiology*.

eTable 1. MAP and VIS During First 12 Hours of CICU Admission According to Randomization Systolic Blood Pressure

**MAP**, median (IQR), mmHg in standard group					
	Q1	Q2	Q3	Q4	*P*-value
CICU admission	76.0 (64.0-86.0)	71.5 (65.2-82.2)	75.5 (64.0-90.0)	75.5 (64.8-91.0)	0.62
1 hour	72.5 (61.2-77.5)	72.0 (64.0-78.8)	75.0 (68.0-84.0)	74.0 (64.2-85.5	0.34
3 hour	71.0 (64.0-80.0)	71.0 (66.0-76.8)	77.5 (69.8-82.0)	75.0 (69.5-82.0)	0.11
6 hour	69.0 (62.5-73.0)	66.5 (64.0-78.5)	75.5 (66.2-88.5)	70.0 (65.5-77.5)	0.09
12 hour	70.0 (64.0-74.0)	72.0 (64.5-75.2)	69.5 (65.2-79.0)	71.0 (66.2-80.0)	0.67
**MAP**, median (IQR), mmHg in microaxial flow pump group					
	Q1	Q2	Q3	Q4	*P*-value
CICU admission	83.0 (72.8-94.8)	78.0 (65.8-94.2)	80.5 (70.8-92.5)	80.5 (68.2-90.0)	0.77
1 hour	75.0 (69.0-89.0)	77.0 (64.5-85.2)	80.0 (68.8-89.5)	80.0 (69.5-93.0)	0.42
3 hour	77.0 (66.2-86.5)	71.0 (59.0-76.5)	77.5 (69.2-89.2)	77.0 (65.5-88.0)	0.09
6 hour	74.0 (66.2-84.2)	70.0 (64.0-83.5)	71.0 (63.5-79.0)	75.0 (67.8-84.0)	0.67
12 hour	74.0 (65.0-84.2)	74.0 (66.0-84.0)	70.5 (65.0-78.0)	73.0 (68.0-83.2)	0.78
**VIS**, median (IQR), in standard group					
	Q1	Q2	Q3	Q4	*P*-value
CICU admission	18.0 (0.0-35.0)	4.8 (0.0-23.0)	2.8 (0.0-17.1)	2.5 (0.0-12.5)	0.16
1 hour	20.0 (4.9-41.2)	10.0 (0.0-28.4)	8.2 (0.0-21.2)	7.0 (0.0-20.0)	0.08
3 hour	21.0 (9.3-40.0)	15.5 (7.0-42.0)	16.0 (0.0-25.4)	12.2 (1.2-20.0)	0.10
6 hour	30.0 (13.0-43.0)	26.0 (14.5-37.2)	15.0 (0.0-26.1)	21.0 (6.0-40.0)	0.06
12 hour	19.0 (12.5-43.8)	18.6 (1.9-31.2)	13.0 (0.0-29.6)	12.0 (3.1-39.6)	0.21
**VIS**, median (IQR) in microaxial flow pump group					
	Q1	Q2	Q3	Q4	*P*-value
CICU admission	5.0 (0.0-13.3)	5.0 (0.0-28.8)	3.0 (0.0-13.3)	0.0 (0.0-12.4)	0.42
1 hour	9.0 (1.0-20.0)	7.0 (0.0-28.8)	6.0 (0.0-21.8)	2.0 (0.0-11.5)	0.24
3 hour	11.2 (5.0-29.0)	6.0 (0.1-30.0)	6.0 (0.0-20.4)	8.3 (0.0-25.9)	0.29
6 hour	11.0 (3.0-33.0)	12.0 (3.0-29.0)	6.8 (0.0-25.2)	9.0 (1.9-22.0)	0.72
12 hour	9.7 (3.9-24.7)	7.6 (3.1-27.0)	13.0 (1.0-30.0)	4.0 (0.5-20.5)	0.47

© 2025 Mikkelsen AD et al. *JAMA Cardiology*.

eTable 2. Incidence of Adverse Events or Death During CICU Stay According to Quartiles of Randomization Systolic Blood Pressure

Abbreviations: CICU, cardiac intensive care unit; Q, quartile; AKI, acute kidney injury; KRT, renal replacement therapy.

		**Q1**	**Q2**	**Q3**	**Q4**
		n = 89	n = 90	n = 90	n = 82
Moderate or severe bleeding or died in CICU, No. (%)	Standard	30 (67)	24 (52)	13 (32)	20 (48)
	mAFP	22 (50)	20 (46)	29 (59)	18 (45)
AKI or died in CICU, No. (%)	Standard	36 (80)	30 (65)	19 (46)	27 (64)
	mAFP	34 (77)	29 (66)	36 (74)	32 (80)
KRT or died in CICU, No. (%)	Standard	32 (71)	24 (52)	15 (37)	24 (57)
	mAFP	26 (59)	27 (61)	33 (67)	23 (58)
Limb ischemia or died in CICU, No. (%)	Standard	28 (62)	22 (48)	12 (29)	19 (45)
	mAFP	22 (50)	15 (34)	22 (45)	15 (38)
Stroke or died in CICU, No. (%)	Standard	28 (62)	23 (50)	13 (32)	19 (45)
	mAFP	22 (50)	15 (34)	24 (49)	14 (35)

© 2025 Mikkelsen AD et al. *JAMA Cardiology*.

eFigure 1. Odds Ratios for 180-Day Mortality by Randomization Systolic Blood Pressure

Odds ratios for 180-day all-cause mortality across a continuum of systolic blood pressure at randomization for the three regression models:

Unadjusted: Crude association

Adjusted: Confounder adjusted model (age, sex, diabetes, hypertension, prior stroke, eGFR at admission, time from symptom onset to randomization, resuscitation prior to arrival in the catheterization laboratory, number of diseased coronary vessels, and randomization group)

Direct: Confounder and mediator adjusted model, evaluating the association of randomization systolic blood pressure and mortality adjusted for the influence of potential downstream mediators of hypoperfusion (lactate, LVEF, TIMI flow post-PCI, blood glucose and SCAI stage)

Shaded areas indicate 95% confidence intervals. The vertical black line indicates the median systolic blood pressure. The dashed red line represents an odds ratio of 1. Bottom grey curve for data density.

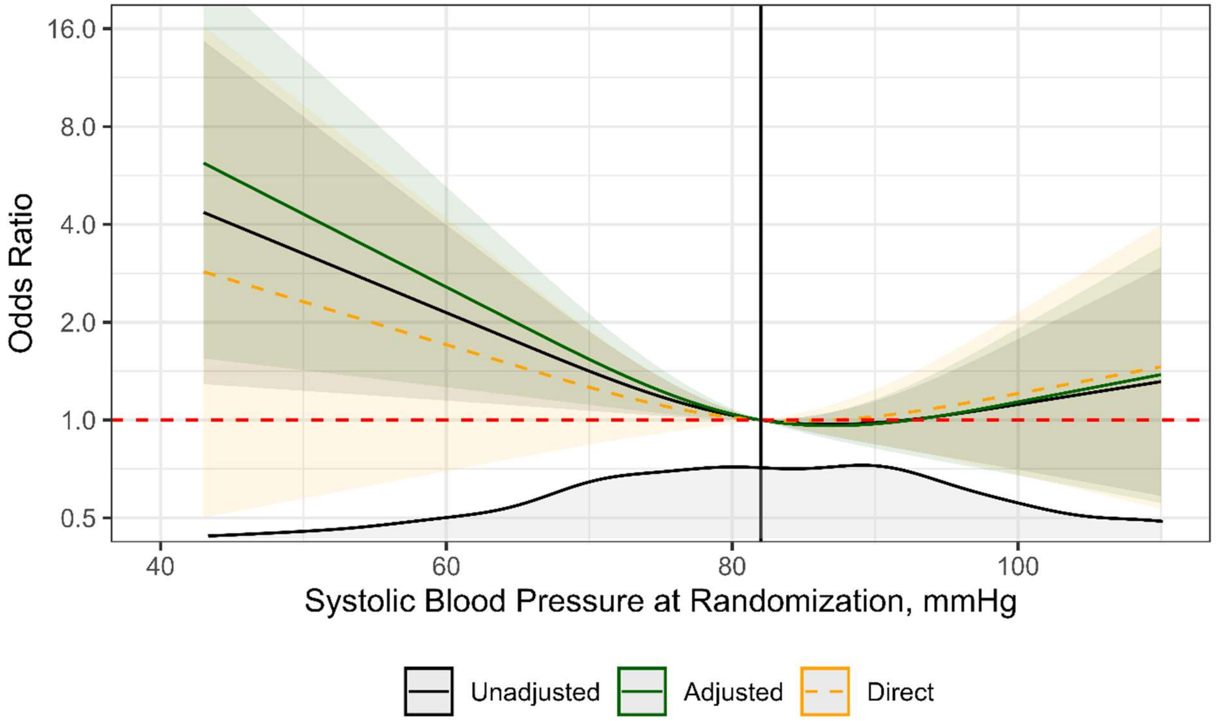

© 2025 Mikkelsen AD et al. *JAMA Cardiology*.

eFigure 2. 180-Day Mortality by Treatment Group Across Mean Arterial Pressure at Randomization

Top panel: mAFP = microaxial flow pump; Standard = standard care alone. Bottom panel: Dashed red line indicates Odds Ratio=1. Shaded areas indicate 95% CI. Bottom grey curve for data density. The model is unadjusted

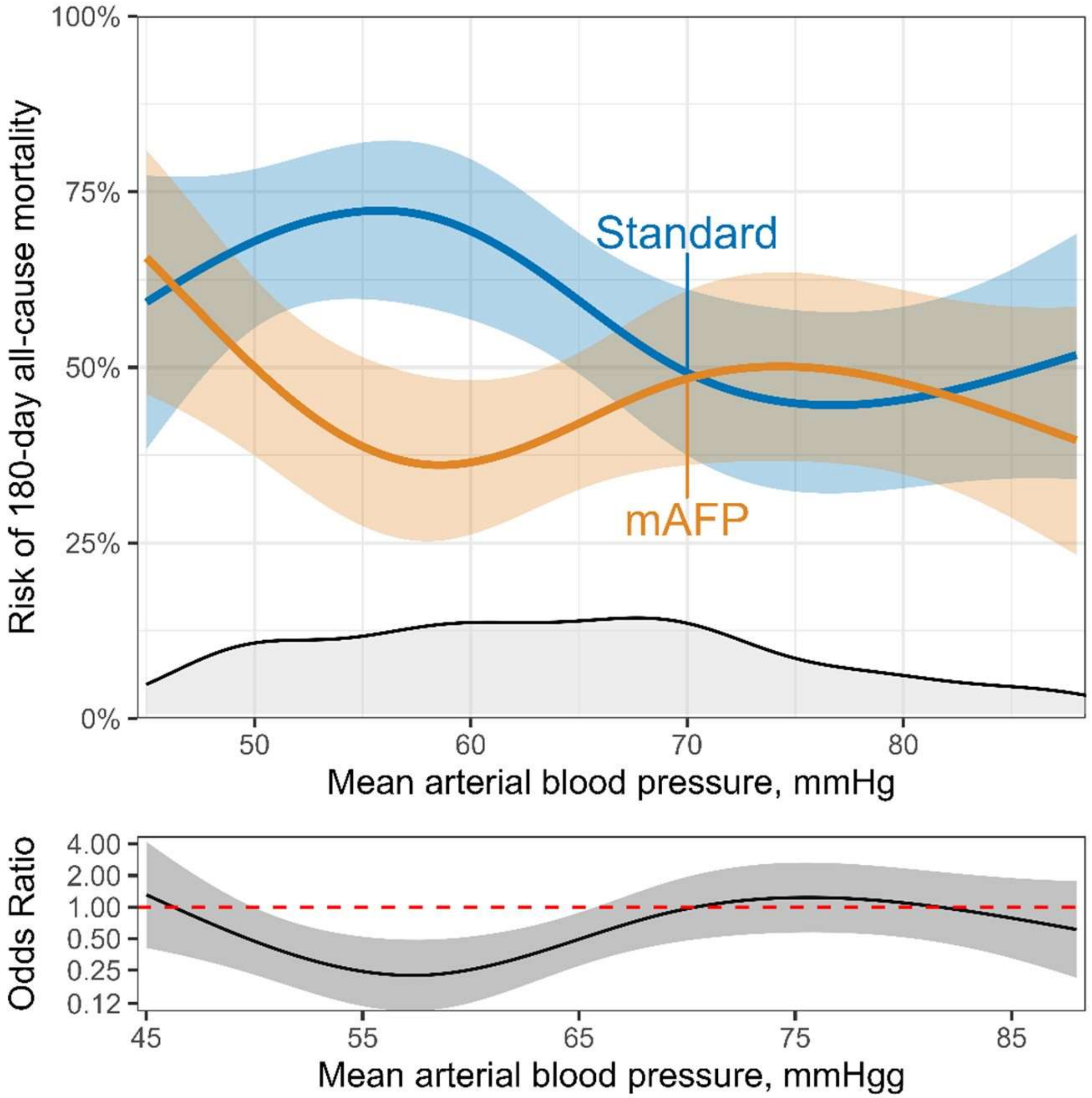

© 2025 Mikkelsen AD et al. *JAMA Cardiology*.
