# Supplementary material for: Systolic Blood Pressure and Microaxial Flow Pump–Associated Survival in Infarct-Related Cardiogenic Shock: A Post Hoc Analysis of the DanGer Shock Randomized Clinical Trial
Source: JAMA Cardiol. 2025 Aug 30;10(11):1157–65. doi: 10.1001/jamacardio.2025.3337 (PMC12398770; doi:10.1001/jamacardio.2025.3337)
Supplement: Supplement 2. — DanGer Shock Investigators [file jamacardiol-e253337-s002.pdf]

Supplemental Online Content: Nonauthor Collaborators
*First name, last name, and suffix (if applicable) are required and will appear in PubMed.

***Group Name(s): DanGer Shock Investigators**

***First Name and Middle Initial(s)**	***Last Name**	***Suffix (eg, Jr, III)**	Academic Degrees	Institution	Location (city, state/province, country)	Role or Contribution, eg, chair, principal investigator	Group (if more than 1 Group listed in the byline) and/or Subgroup (eg, Steering Committee)
Jacob Eifer	Møller		MD, DMSc	Odense University Hospital / University of Southern Denmark	Odense, Denmark	Principal Investigator and Sponsor	
Lisette Okkels	Jensen		MD, DMSc	Odense University Hospital	Odense, Denmark		
Anders	Junker		MD, PhD	Odense University Hospital	Odense, Denmark		
Karsten Tange	Veien		MD	Odense University Hospital	Odense, Denmark		
Nanna Louise Junker	Udesen		MD, PhD	Odense University Hospital	Odense, Denmark		
Henrik	Schmidt		MD, DMSc	Odense University Hospital	Odense, Denmark		
Christian	Hassager		MD, DMSc	Copenhagen University Hospital Rigshospitalet	Copenhagen, Denmark	PI	
Thomas	Engstrøm		MD, DMSc	Copenhagen University Hospital Rigshospitalet	Copenhagen, Denmark		
Lene	Holmvang		MD, DMSc	Copenhagen University Hospital Rigshospitalet	Copenhagen, Denmark		
Jesper	Kjærgaard		MD, DMSc	Copenhagen University Hospital Rigshospitalet	Copenhagen, Denmark		
Rikke	Sørensen		MD, PhD	Copenhagen University Hospital Rigshospitalet	Copenhagen, Denmark		
Jacob	Lønborg		MD, DMSc	Copenhagen University Hospital Rigshospitalet	Copenhagen, Denmark		
Martin	Frydland		MD, PhD	Copenhagen University Hospital Rigshospitalet	Copenhagen, Denmark		
Rasmus Paulin	Beske		MD, PhD	Copenhagen University Hospital Rigshospitalet	Copenhagen, Denmark		
Søren	Boesgaard		MD, DMSc	Copenhagen University Hospital Rigshospitalet	Copenhagen, Denmark		
Hans	Eiskjær		MD, DMSc	Aarhus University Hospital	Aarhus, Denmark	PI	
Steffen	Christensen		MD, PhD	Aarhus University Hospital	Aarhus, Denmark		
Evald Høj	Christiansen		MD, PhD	Aarhus University Hospital	Aarhus, Denmark		
Christian Juhl	Terkelsen		MD, DMSc	Aarhus University Hospital	Aarhus, Denmark		
Andreas	Schäfer		MD	Hannover Medical School	Hannover, Germany	National German PI	
Axel	Linke		MD, DMSc	Heart Center Dresden / TU Dresden	Dresden, Germany	PI	

Supplemental Online Content: Nonauthor Collaborators
*First name, last name, and suffix (if applicable) are required and will appear in PubMed.

***First Name and Middle Initial(s)**	***Last Name**	***Suffix (eg, Jr, III)**	Academic Degrees	Institution	Location (city, state/province, country)	Role or Contribution, eg, chair, principal investigator	Group (if more than 1 Group listed in the byline) and/or Subgroup (eg, Steering Committee)
Felix J	Woitek		MD	Heart Center Dresden / TU Dresden	Dresden, Germany		
Jennifer	Hommel		PhD	Heart Center Dresden / TU Dresden	Dresden, Germany		
Norman	Mangner		MD	Heart Center Dresden / TU Dresden	Dresden, Germany		
Amin	Polzin		MD	University Hospital Düsseldorf	Düsseldorf, Germany	PI	
Ralf	Westenfeld		MD	University Hospital Düsseldorf	Düsseldorf, Germany		
Christian	Schulze		MD	University Hospital Jena	Jena, Germany	PI	
Sven	Moebius-Winkler		MD, PhD	University Hospital Jena	Jena, Germany		
Carsten	Skurk		MD	Deutsches Herzzentrum der Charité	Berlin, Germany	PI	
Peter	Nordbeck		MD	University Hospital Würzburg	Würzburg, Germany	PI	
Peter	Clemmensen		MD, DMSc	University Heart and Vascular Center Hamburg	Hamburg, Germany	PI	
Dirk	Westermann		MD, PhD	University Heart and Vascular Center Hamburg	Hamburg, Germany		
Vasileios	Panoulas		MD	Royal Brompton and Harefield Hospitals	Harefield, United Kingdom	PI	
Sebastian	Zimmer		MD	University Hospital Bonn	Bonn, Germany	PI	
Nikos	Werner		MD, PhD	Heartcenter Trier	Trier, Germany	PI	
Kristian	Wachtell		MD, DMSc	Odense University Hospital	Odense, Denmark		
Hanne Berg	Ravn		MD, DMSc	Odense University Hospital	Odense, Denmark		
Jens Flensted	Lassen		MD, DMSc	Odense University Hospital	Odense, Denmark		
Inge	De Haas		MD	Aalborg University Hospital	Aalborg, Denmark		
Oke	Gerke		Cand.scient, PhD	Odense University Hospital / University of Southern Denmark	Odense, Denmark	Statistician	
