# Supplementary material for: Systolic Blood Pressure and Microaxial Flow Pump–Associated Survival in Infarct-Related Cardiogenic Shock: A Post Hoc Analysis of the DanGer Shock Randomized Clinical Trial
Source: JAMA Cardiol. 2025 Aug 30;10(11):1157–65. doi: 10.1001/jamacardio.2025.3337 (PMC12398770; doi:10.1001/jamacardio.2025.3337)
Supplement: Supplement 3. — Data Sharing Statement [file jamacardiol-e253337-s003.pdf]

# Data Sharing Statement

Mikkelsen. Systolic Blood Pressure and Microaxial Flow Pump–Associated Survival in Infarct-Related Cardiogenic Shock. *JAMA Cardiol*. Published August 30, 2025.
doi:10.1001/jamacardio.2025.3337

**Data**
**Additional Information:** ClinicalTrials.gov,NCT01633502 (https://clinicaltrials.gov/ct2/show/NCT01633502)
**Data available:** No

**Additional Information**
**Explanation for why data not available:** Data sharing may be possible based on a reasonable request (e.g., for individual patient data metaanalyses).
